# Analysis of patient reported outcomes included in the registrational clinical trials of nivolumab for advanced non-small cell lung cancer

**DOI:** 10.1016/j.tranon.2022.101418

**Published:** 2022-04-13

**Authors:** Remziye Zaim, Ken Redekop, Carin A. Uyl-de Groot

**Affiliations:** Erasmus School of Health Policy & Management, Erasmus University Rotterdam, Rotterdam, the Netherlands

**Keywords:** Patient reported outcomes, Nivolumab, Non-small cell lung cancer, Health-related quality of life, Immune-related adverse event, Ipilimumab

## Abstract

In the era of value-based oncology care, stakeholders are increasingly using patient reported outcomes (PROs) to guide clinical and regulatory decisions. PROs are also included in health technology assessments to guide patient access, drug reimbursement and pricing. We reviewed PROs collected in the United States Food and Drug Administration approved indications of nivolumab in advanced NSCLC. We analyzed the PRO data reported in the CheckMate 9LA (NCT03215706), CheckMate 227 (NCT02477826), CheckMate 057 (NCT01673867), and CheckMate 017 (NCT01642004) registrational clinical trials, and concluded that nivolumab alleviated symptom burden and improved health status of patients in this setting. However, inability of the included PRO instruments to measure immune-related adverse events, differences in the timing of PRO evaluation between treatment groups, incomplete patient participation at all time points, limited patient participation in the later time points, and interpretation of the longitudinal data are key challenges that impede accurate analysis and validation of PROs.

## Highlights

• Patients’ perspectives are at the center of value-based oncology care.
• Patient reported outcomes (PROs) guide clinical and regulatory decisions.
• PRO instruments do not capture immune-related adverse events in clinical trials.
• Studies did not accurately report PROs after treatment discontinuation.
• Precise analyses of longitudinal effects of nivolumab on PROs were lacking.

## Introduction

Immune checkpoint blockade is an effective therapeutic strategy that harnesses the immune system to generate an antitumor response. [1] Nivolumab, a programmed cell death receptor-1 (PD-1) blocking antibody, prolongs survival alone or in combination with ipilimumab, a cytotoxic T lymphocyte antigen-4 (CTLA-4) receptor, in the treatment of metastatic or recurrent non-small cell lung cancer (NSCLC). [2], [3], [4], [5], [6] The US Food and Drug Administration (FDA) approved nivolumab for three NSCLC indications: [2]

1 adult patients with metastatic NSCLC expressing PD Ligand-1 (PD-L1) (≥1%) as determined by an FDA-approved test, [7] with no epidermal growth factor receptor (EGFR) or anaplastic lymphoma kinase (ALK) genomic tumor aberrations, as first-line treatment in combination with ipilimumab.
2 adult patients with metastatic or recurrent NSCLC with no EGFR or ALK genomic tumor aberrations as first-line treatment, in combination with ipilimumab and two cycles of platinum-doublet chemotherapy.
3 patients with metastatic NSCLC and progression on or after platinum-based chemotherapy. Patients with EGFR or ALK genomic tumor aberrations should have disease progression on FDA-approved therapy for these aberrations prior to receiving nivolumab.

Approval of these three indications by the US FDA were based on enhanced efficacy and acceptable toxicity profiles of nivolumab, investigated in randomized, open-label, Phase III clinical trials. [3], [4], [5], [6] The 21st Century Cures Act [8] outlines ways for the US FDA to incorporate patients’ experience into drug development and review processes, and initiatives spearheaded by the US FDA patient-focused drug development program. [9] There is evidence that monitoring treatment side effects in real time can improve outcomes for patients with cancer, including a potential benefit in survival rates. [10] Previous research has shown that PRO data captured during treatment may increase accuracy in the assessment of patients’ experience of symptomatic side effects compared with clinician reports, because clinicians may underreport the frequency or severity of side effects. [11] NSCLC is classified as a high tumor mutational burden (TMB) cancer. [12] During or after immunotherapy, patients may experience immune-related adverse events (irAEs), in addition to commonly reported treatment-related side effects. [12] Although patients’ assessments of the incidence and consequences of these irAEs are important, existing cancer-specific PRO instruments [13,14] were not designed to capture irAEs, and may not fully reflect the benefits and toxicity profiles of immunotherapies, such as nivolumab and ipilimumab. Guidelines for reporting clinical trials promote transparent and accurate reporting of PROs, in an effort to facilitate interpretation of these complex data and their limitations, which are further compounded by factors such as the unblinded nature of the NSCLC clinical trials. [15,16]

Value frameworks encompassing benefits, toxicity, and costs of medical technologies can be used to quantify the net value of NSCLC therapies; enabling comparisons, formulary prioritization, and cost-effectiveness assessments. For example, the value framework used by the National Institute for Health and Care Excellence and the Institute for Clinical and Economic Review includes quality-adjusted life years (QALY). [17], [18], [19] Similarly, the European Society for Medical Oncology enables optional weighting of efficacy outcomes based on health-related quality of life (HRQoL). [20] Therefore, PROs are increasingly included in health technology assessments, and have important ramifications on patient access, drug reimbursement and pricing. The importance of collecting appropriate PROs is also reflected in the updated US FDA and European Medicines Agency (EMA) drug approval processes. [21,22] In this study, we aim to analyze PRO data reported in the registrational clinical trials of nivolumab in metastatic or recurrent NSCLC, and assess whether these data were collected rigorously using appropriate, reliable, and validated instruments.

## Extraction of PRO data

We reviewed registration clinical trials of nivolumab in the US FDA databases and Clinicaltrials.gov, from January 1, 2012 until January 1, 2022. Published studies reporting PROs from the registrational trials of nivolumab were also searched using PubMed. (See *Supplementary Appendix*) Study findings were summarized descriptively. In addition, clinical trial number, approval date by the US FDA, publication author and year, trial phase, treatment(s), comparator(s), number of patients available for analysis, PRO instruments used in the trial, PRO assessment frequency, PRO completion rates, and PRO follow-up after treatment discontinuation were presented in tabular format.

## Analysis of PRO data

PRO data have been included as exploratory and/or secondary endpoints in the included nivolumab clinical trials. The PROs used in nivolumab clinical trials included a generic HRQoL measure, the EuroQoL five dimensions (EQ-5D) 3-level version, [23,24] as well as a tumor-specific measure, the Lung Cancer Symptom Scale (LCSS). [14] LCSS includes the average symptom burden index ([ASBI]; based on six symptoms: anorexia, fatigue, cough, dyspnea, hemoptysis, and pain) and the 3-item global index ([3-IGI]; symptom distress, interference with activities, and HRQoL). [25] EQ-5D includes the utility index [UI] and visual analog scale [VAS]. The EQ-5D descriptive index responses were mapped into UI ranging from death (0) to full health (1), with health states worse than death being possible (<0), by using utility weights for the United Kingdom (UK) population. Population norms for the UK are 82.8 (EQ-5D VAS) and 0.86 (EQ-5D UI) [26]; published estimates for patients with lung cancer in the UK are 68 (EQ-5D VAS) and 0.67 (EQ-5D UI). [27] Table 1 shows the details of the PRO instruments. In the published studies, PRO data were analyzed using descriptive statistics within each treatment arm, comparing scores during treatment to baseline scores and between treatment arms at specific time points. Longitudinal changes from baseline within and between arms were assessed with mixed-effects models for repeated measures (MMRM). Time to deterioration or improvement in HRQoL, defined based on clinically meaningful change in score, was determined using Kaplan-Meier method. A clinically meaningful change in score represents a treatment benefit or harm perceptible by the patient, and significant enough to warrant a modification to the patient's clinical management. Changes in scores are also interpreted relative to the minimally important difference (MID), which is the smallest difference in score that patients perceive as beneficial or detrimental, and is established by extensive anchor-based and/or distribution-based quantitative analyses. [27], [28], [29], [30] A MID was defined as a within-patient score difference between baseline and a given time point of 10 points for the LCSS [ASBI] and 30 points for the LCSS [3-IGI]. [14] A MID was defined as a score difference of 0.08 points for the EQ-5D [UI] and 7 points for the EQ-5D [VAS]. [27]

**Table 1:** PRO instruments used in the registrational clinical trials of nivolumab for metastatic or recurrent NSCLC.

	**CheckMate 9LA [****31****]**	**CheckMate 227 [****32****]**	**CheckMate 057 [****33****]**	**CheckMate 017 [****34****]**
Trial Number	NCT03215706	NCT02477826	NCT01673867	NCT01642004
Trial Phase	Phase III trial, randomized, open-label	Phase III trial, randomized, open-label	Phase III trial, randomized, open-label	Phase III trial, randomized, open-label
US FDA approval date	May 26, 2020	May 15, 2020	October 9, 2015	March 4, 2015
Publication Author, Year	Abstract only; Reck M, et al. 2020	Reck M, et al. 2021	Reck M, et al. 2018	Reck M, et al. 2018
Patients	Treatment naive, stage IV or recurrent NSCLC, and no known sensitizing EGFR/ALK alterations	Treatment naïve, advanced NSCLC with ≥1% PD-L1, and high TMB (≥10 mutations per mega base)	Non-squamous advanced NSCLC patients with disease progression during or after platinum doublet chemotherapy	Squamous advanced NSCLC patients with disease progression during or after one platinum doublet chemotherapy
Treatment(s)	Nivolumab (360 mg Q3W) + Ipilimumab (1 mg/kg Q6W) + 2 cycles of chemotherapy *(N = 361)*	Nivolumab (3 mg/kg Q2W) + Ipilimumab (1 mg/kg Q6W), *(N = 396)*	Nivolumab (3 mg/kg Q2W), *(N = 292)*	Nivolumab (3 mg/kg Q2W), *(N = 135)*
Comparator(s)	4 cycles of chemotherapy *(N = 358)*	Nivolumab monotherapy, or Platinum doublet chemotherapy⁎, *(N = 397)*	Docetaxel (75 mg/m^2^ Q3W), *(N = 290)*	Docetaxel (75 mg/m^2^ Q3W), *(N = 137)*
PRO Instruments	LCSS [ASBI] and LCSS [3-IGI], EQ-5D-3 L [UI] and EQ-5D-3 L [VAS]	LCSS [ASBI] and LCSS [3-IGI], EQ-5D-3 L [UI] and EQ-5D-3 L [VAS]	LCSS [ASBI] and LCSS [3-IGI], EQ-5D-3 L [UI] and EQ-5D-3 L [VAS]	LCSS [ASBI] and LCSS [3-IGI], EQ-5D-3 L [UI] and EQ-5D-3 L [VAS]
PRO Trial Endpoint	Exploratory	Exploratory	The proportion of pts with disease-related symptom improvement at 12 wk on the LCSS [ASBI] was a secondary endpoint. Overall health status, measured by the EQ-5D-3 L, was an exploratory endpoint.	The proportion of pts with disease-related symptom improvement at 12 wk on the LCSS [ASBI] (*a* ≥ 10-point) was a secondary endpoint. Overall health status, measured by EQ-5D-3 L was an exploratory endpoint.
PRO Assessment Frequency	Not reported in the abstract	#For the first 6 mo of treatment, LCSS and EQ-5D assessments were completed Q2W) for nivolumab plus ipilimumab and Q3W for chemotherapy; beyond 6 mo, these were completed Q6W for both groups while patients were receiving treatment.	Baseline, on day 1 of every other cycle (i.e., every 4 wk) of nivolumab or every cycle (i.e., every 3 wk) of docetaxel for the first 6 mo on treatment before any clinical activities occurred and subsequently, every 6 wk during therapy and at two follow-up visits after treatment discontinuation; EQ-5D assessments continued every 3 mo for 12 mo and then every 6 mo thereafter.	Baseline, on day 1 of every other cycle (i.e., every 4 wk) of nivolumab or every cycle (i.e., every 3 wk) of docetaxel for the first 6 mo on treatment before any clinical activities occurred and subsequently, every 6 wk during therapy and at two follow-up visits after treatment discontinuation; EQ-5D assessments continued every 3 mo for 12 mo and then every 6 mo thereafter.
PRO Completion Rate	> 80% across arms for most on-treatment assessment time points in which there were ≥ 10 pts (up to wk 90 for Nivolumab + Ipilimumab + chemotherapy and wk 78 for chemotherapy)	> 80%	LCSS completion rates at baseline were 82.2% for nivolumab and 76.6% for docetaxel. The EQ-5D completion rates were 83.6% for nivolumab and 80.0% for docetaxel, respectively. At baseline and at one or more post-baseline visits, the rates were: 70.5% (LCSS) and 71.2% (EQ-5D) for nivolumab, 69.7% (LCSS) and 73.1% (EQ-5D) for docetaxel.	LCSS completion rates at baseline were 77.8% for nivolumab and 76.6% for docetaxel. At baseline and at one or more post-baseline visits, the rates were: 68.9% for nivolumab and 62.8% for docetaxel. In both treatment groups, EQ-5D completion rates were >70% up to wk 12.
PRO Follow-up After Treatment Discontinuation	Not reported in the abstract	LCSS and EQ-5D-3 L were completed at two follow-up§ visits after treatment discontinuation. Only EQ-5D-3 L continued every 3 mo for 12 mo, and then every 6 mo thereafter, at survival.†	LCSS and EQ-5D-3 L were completed at two follow-up^§^ visits after treatment discontinuation. Only EQ-5D-3 L continued every 3 mo for 12 mo, and then every 6 mo thereafter, at survival.†	LCSS and EQ-5D-3 L were completed at two follow-up^§^ visits after treatment discontinuation. Only EQ-5D-3 L continued every 3 mo for 12 mo, and then every 6 mo thereafter, at survival.†

⁎ Chemotherapy was dependent on tumor histology and administered every 3 weeks for up to four cycles, with optional pemetrexed maintenance for patients with non-squamous NSCLC. Immunotherapy continued until disease progression, unacceptable toxicity, or for 2 years.

# Common time points to both treatment groups were at 6-week intervals. LCSS and EQ-5D were administered at follow-up visits 1 and 2. EQ-5D was also administered at survival follow-up visits (every 3 mo for the first year and then every 6 mo).

§ Follow-up visit 1 occurred 35 (±7) days from the last dose or at treatment discontinuation (±7 days), if the date of discontinuation was greater than 42 days from the last dose; follow-up visit 2 occurred 80 (±7) days from follow-up visit 1.

† Survival follow-up visits occurred approximately every 3 months (±7 days) from follow-up visit 2. PRO: Patient Reported Outcome; NSCLC: Non-small Cell Lung Cancer; LCSS: Lung Cancer Symptom Scale; ASBI: Average Symptom Burden Index; 3-IGI: 3-Item Global Index; EQ5D-3L: EuroQoL 5-dimensional instrument- 3 Level; UI: Utility Index; VAS: Visual analog scale; TMB: Tumor Mutational Burden; PD-L1: Programmed Cell Death-Ligand 1; EGFR: Epidermal Growth Factor Receptor; ALK: Anaplastic Lymphoma Kinase; QW: every week, mo: month; w: week; d: day; kg: kilogram; m^2^: meter square; pts: patients.

## Registrational clinical trials of nivolumab for treatment naïve NSCLC patients

In the CheckMate 9LA trial, [6] PROs were exploratory endpoints; disease-related symptoms were evaluated using the LCSS ASBI and 3-IGI; HRQoL was evaluated using EQ-5D-3 L UI and VAS. Analyses included mean changes from baseline, MMRM of longitudinal changes, and TTD. PRO completion rates were > 80% across arms for most on-treatment assessment time points in which there were ≥ 10 patients (up to week 90 for nivolumab plus ipilimumab plus chemotherapy, and week 78 for chemotherapy). [31] PRO follow-up after treatment discontinuation was not reported in the study abstract. A trend for improvement in LCSS ASBI and 3-IGI was reported in both treatment arms, however, the MID was not reached. In both arms, mean EQ-5D-3 L VAS scores approached the UK population norms after about 30 weeks. MMRM analyses showed similar improvement across arms in overall LCSS ASBI, when there was a sufficient number of patients in both study arms for assessment (up to week 78). [31] There was a decreased risk of, and delayed time to, definitive deterioration with nivolumab plus ipilimumab plus chemotherapy versus chemotherapy. Time from randomization to definitive deterioration (all subsequent assessments that met/exceeded the deterioration threshold) were HR (95% CI); LCSS ASBI 0.66 (0.47–0.92), LCSS 3-IGI 0.66 (0.50–0.88), EQ-5D-3 L VAS 0.73 (0.58–0.93), EQ-5D-3 L UI 0.72 (0.57–0.90). [31] Based on the limited data obtained from the published abstract, patients with advanced NSCLC treated with nivolumab plus ipilimumab plus chemotherapy (2 cycles) maintained their quality of life as compared with chemotherapy (4 cycles). Patients in the nivolumab plus ipilimumab plus chemotherapy arm had decreased risk of definitive deterioration in HRQoL and symptoms compared with chemotherapy.

In the CheckMate 227 trial, [5] PROs were assessed as an exploratory endpoint; disease-related symptoms were evaluated using the LCSS ASBI and 3-IGI; HRQoL was evaluated using EQ-5D-3 L UI and VAS. PRO analysis included patients with high TMB (≥10 mutations/mega base). [32,35] PROs were evaluated each cycle (Q2W, nivolumab plus ipilimumab; Q3W, chemotherapy) for the first 6 months, every 6 weeks thereafter during treatment, and at follow-up visits 1 and 2. Only EQ-5D-3 L was assessed during survival follow-up. Longitudinal changes from baseline were assessed by MMRMs and TTD analyses. PRO completion rates were >80% for most on-treatment assessments. [32,35] The mean baseline scores (95% CI) for nivolumab plus ipilimumab and chemotherapy, were LCSS ASBI, 27.7 (24.6–30.8) and 24.8 (22.2–27.5); LCSS 3-IGI, 195.8 (183.0–208.6) and 197.6 (185.4–209.8); fatigue, 35.8 (31.2–40.4) and 36.0 (31.5–40.5); dyspnea, 28.8 (23.9–33.8) and 24.8 (20.4–29.1), respectively. [32,35] Differences in mean changes from baseline in LCSS 3-IGI favored nivolumab plus ipilimumab versus chemotherapy, with the difference being higher than the MID for the overall score (mean change 27.5 versus −5.1; difference 32.6) and higher than or approaching the MID for individual items. Similarly, differences in EQ-5D VAS and EQ-5D UI mean scores, and changes from baseline favored nivolumab plus ipilimumab versus chemotherapy, mean scores for these measures approaching the general population scores in the UK. [32,35] In the CheckMate 227 trial, the magnitude of difference was small for EQ-5D VAS; but for EQ-5D UI, differences were clinically meaningful (difference in least squares mean change of 0.091). TTD by LCSS ASBI and by LCSS 3-IGI were delayed with nivolumab plus ipilimumab, with HRs for nivolumab plus ipilimumab over chemotherapy of 0.40 (95% CIs; 0.26–0.63) and 0.56 (0.38–0.82), respectively. [32,35] However, patients without significant deterioration and significant improvement at 1-year was only 10% better with the immunotherapy combination. [32,35] EQ-5D VAS and UI results were (95% CI) 0.62 (0.42–0.92) and 0.50 (0.34–0.73), respectively. [32,35] In both treatment groups, after treatment discontinuation (follow-up visits 1 and 2), mean changes from baseline in LCSS ASBI, LCSS 3-IGI, EQ-5D UI and EQ-5D VAS scores were small. All in all, in the CheckMate 227 trial, nivolumab plus ipilimumab delayed deterioration in symptoms, and improved HRQoL compared to chemotherapy in advanced NSCLC patients with 1% or greater PD-L1 expression.

## Registrational clinical trials of nivolumab for previously treated NSCLC patients

In the CheckMate 057 trial, [3] the proportion of patients with disease-related symptom improvement at 12 weeks on the LCSS was a secondary endpoint. Overall health status, measured by the EQ-5D UI and VAS, was an exploratory endpoint. PROs were evaluated each cycle for the first 6 months, every 6 weeks thereafter during treatment, and at follow-up visits 1 and 2. Only EQ-5D-3 L was assessed during survival follow-up. The questionnaire completion rates were generally similar for nivolumab versus docetaxel at baseline (EQ-5D: 84% vs. 80%; LCSS: 82% vs. 77%), and at week 12 (EQ-5D: 77% vs. 80%; LCSS: 77% vs. 76%). [33] MMRM analyses showed that the differences in mean changes from baseline (95% CI) for the LCSS ASBI and 3-IGI were −5.8 (−8.5 to −3.0) and 20.3 (9.6–31.0), respectively. [33] For the EQ-5D UI and VAS, the results were (95% CI) 0.034 (−0.009 to 0.076) and 5.9 (2.2–9.7), respectively. [33] TTD analyses for the LCSS ASBI and 3-IGI were (95% CI) 0.65 (0.49–0.85) and 0.63 (0.48–0.82), respectively. [33] For the EQ-5D UI and VAS, the results were (95% CI) 0.90 (0.69–1.17) and 0.76 (0.59–0.98), respectively. [33] Mean baseline LCSS ASBI scores were similar in both arms. The proportion of patients with disease-related symptom improvement (95% CI) by week 12 was 17.8% (13.6–22.7) with nivolumab, and 19.7% (15.2–24.7) with docetaxel. [33] LCSS ASBI scores improved with nivolumab and worsened with docetaxel at weeks 12, 24, 30 and 42. In the CheckMate 057 trial, nivolumab improved disease-related symptoms and overall health status compared to docetaxel for the second-line treatment of advanced non-squamous NSCLC.

In the CheckMate 017 trial, [4] the proportion of patients with disease-related symptom improvement at 12 weeks on the LCSS was a secondary endpoint. Overall health status, measured by the EQ-5D UI and VAS, was an exploratory endpoint. PROs were evaluated each cycle for the first 6 months, every 6 weeks thereafter during treatment, and at follow-up visits 1 and 2. Only EQ-5D-3 L was assessed during survival follow-up. MMRM analyses showed that the differences in mean changes (95% CI) for the LCSS ASBI and 3-IGI were −5.6 (95% CI; −10.5 to −0.6) and 22.2 (2.5 - 41.8), respectively. [34] For the EQ-5D UI and VAS, the results were (95% CI) 0.027 (−0.047 to 0.100) and 7.2 (0.6 to 13.8), respectively. [34] TTD analyses for the LCSS ASBI and 3-IGI were (95% CI) 0.67 (95% CI; 0.43–1.03) and 0.57 (0.38–0.85). For the EQ-5D UI and VAS, the results were (95% CI) 0.55 (0.36–0.84) and 0.59 (0.40–0.87), respectively. [34] The mean EQ-5D UI scores in the nivolumab group were more favorable than the mean score of a general population in the United States (US) (0.87) [36] beginning at week 42, whereas the scores in the docetaxel group were similar to the norm for a population with lung cancer (0.67). [27] The mean VAS scores in the nivolumab group exceeded the US general population norm (80.05) [36] at weeks 48 and 60, but the scores in the docetaxel group were comparable with those in a population with lung cancer (68). [27] After treatment discontinuation (follow-up visits 1 and 2), estimated changes from baseline in the LCSS ASBI and 3-IGI scores showed worsening in both treatment groups. For the ASBI, the estimated changes (range 5.5–9.5 points) were less than the MID and were significant only in the docetaxel group. [34] For the 3-IGI, there was significant worsening in the nivolumab (follow-up visit one only) and docetaxel groups (both follow-up visits), with estimated changes greater than the MID in the docetaxel group. There were no significant between-treatment group differences with either instrument after treatment discontinuation. In the CheckMate 017 trial, nivolumab alleviated symptom burden and improved health status compared to docetaxel in the treatment of second-line squamous NSCLC.

## Discussion

PRO data reported across all registrational clinical trials of nivolumab in NSCLC indicated that treatment with nivolumab stabilizes or improves HRQoL, and alleviates symptom burden while providing clinical benefits. Despite the recognition that alleviating symptom burden and improving HRQoL are critical components of cancer care, particularly for highly symptomatic tumors such as advanced NSCLC, accurate studies of PROs included in the clinical trials are limited. Overall findings from CheckMate 9LA, CheckMate 227, CheckMate 057 and CheckMate 017 suggest treatment with nivolumab is favorable. However, these findings should be interpreted with caution, specifically, for the CheckMate 9LA trial, because a detailed PRO analysis has not been published at the time of our analysis. Similar to nivolumab, ipilimumab's mechanism of action relies on the generation of a T cell-mediated immune antitumor response. While irAEs are very common in patients treated with immunotherapies, particularly with an anti-CTLA-4 antibody, the PRO instruments included in the CheckMate 9LA and CheckMate 227 trials did not have the capabilities to measure consequences of irAEs on the patients. In the CheckMate 227 trial, clinical outcomes of nivolumab plus ipilimumab was compared with nivolumab alone, however the PRO analyses did not include this important comparison for further insights. It would have been informative to assess whether a correlation (or lack thereof) was observed for PRO benefits and progression-free or overall survival. [37]

In general, several studies have raised concerns about PRO assessments in randomized clinical trials. [38,39] There have been concerns on reporting bias when measuring PROs in open-label trials, although some of these concerns have been challenged. [40,41] Given that patients may have increased expectations about the nivolumab, open-label studies may compel more patients to complete questionnaires and/or positively rank nivolumab. [33] Conversely, exclusion of patients who discontinued therapy in the on-treatment analyses may lead to an understatement in the difference between the two arms, as discontinuing patients generally represent those with the worst quality of life. [3] As observed in the nivolumab trials, PROs are often included as an exploratory endpoint without a rationale or a hypothesis on the expected benefit. Moreover, the choice of tools to assess PROs is not always justified. Cancer-specific tools currently used have not been validated to evaluate PROs in patients receiving immunotherapies, and for those experiencing irAEs. This limitation has led to the development of tools such as the Functional Assessment of Cancer Therapy-Immune Checkpoint Modulator. [42] For many of the PRO assessments, the outcomes for patients treated with immunotherapy combination and chemotherapy overlap for many weeks. Also, immunotherapy may produce sustained clinical benefit for some patients in the long-term. Therefore, the calculation of proportional hazards may not adequately reflect delayed benefits from immunotherapy. Modified approaches such as milestone survival analysis have been proposed to quantify the long-term benefits of immunotherapy. [43,44] Such approaches may also be necessary to appropriately evaluate changes in PRO data with immunotherapy. An accurate assessment of PROs with tools that can capture unique therapeutic benefits and toxicities of immunotherapy is needed not only to provide a comprehensive assessment of novel therapies, but also to facilitate application of these tools in clinical practice.

Wang et al. [12] conducted a meta-analysis to demonstrate that irAEs could predict the efficacy of ICIs in lung cancer patients. Authors searched literature to obtain data on objective response rate (ORR), overall survival (OS), or progression-free survival (PFS). A total of 34 records were examined. [12] The irAEs occurrence was significantly associated with higher ORR {risk ratio (RR): 2.43, 95% confidence interval (CI) [2.06–2.88]}, and improved OS {hazard ratio (HR): 0.51, 95% CI [0.43–0.61]}, and PFS (HR: 0.50, 95% CI [0.44–0.57]) in lung cancer patients undergoing ICIs. [12] Subgroup analysis revealed that OS was significantly longer in patients who developed dermatological (OS: HR: 0.53, 95%CI [0.42–0.65]), endocrine (OS: HR: 0.55, 95%CI [0.45–0.67]), and gastrointestinal irAEs (OS: HR: 0.58, 95%CI [0.42–0.80]) than in those who did not. [12] However, hepatobiliary, pulmonary, and high-grade (≥3) irAEs were not correlated with increased OS and PFS. [12] The authors concluded that the occurrence of irAEs in lung cancer patients, particularly dermatological, endocrine, and gastrointestinal irAEs, is a predictor of enhanced ICIs efficacy.

Boutros et al. [45] conducted a systematic review and meta-analysis to compare PROs between ICIs (or ICIs plus chemotherapy) with standard chemotherapy in patients with advanced solid tumors. ICIs were associated with higher levels of QoL and longer time to clinical deterioration on several PRO scales compared with chemotherapy in different types of solid tumors. The co-primary endpoints were time from baseline to first deterioration in PROs, defined as the time from baseline to the first clinically significant deterioration in PROs, and the changes in PROs from baseline to follow-up between ICI and chemotherapy treatment groups. [45] 17 randomized trials of ICIs versus chemotherapy were included in the analysis. Treatment with ICI delayed clinical deterioration over standard chemotherapy in Global Health Status/QoL EORTC QLQ-C30 (hazard ratio [HR] 0.81; 95% confidence interval [CI], 0.74–0.89), and in both EQ-5D utility index (HR 0.65; 95% CI, 0.52–0.82) and EQ-5D visual analogue scale (VAS; HR 0.70; 95% CI, 0.61–0.80). [45] The difference in mean change between the ICI-treated group and the chemotherapy-treated group was 5.82 (95% CI, 4.11–7.53), in favor of ICIs. [45] Similarly, in the EQ-5D, the mean change differences favored treatment with ICIs in both utility index and VAS, with differences of 0.05 (95% CI, 0.03–0.07) and 5.41 (95% CI, 3.39–7.43), respectively. [45]

Similarly, Gonzalez et al. [46] conducted a meta-analysis to quantitatively summarize QOL in patients treated with ICIs. For global QOL, authors used the EORTC QLQ-C30 global health status score, and the EQ-5D visual analog scale. The co-primary endpoints were change in global QOL among patients treated with ICIs and difference in change from baseline in global QOL, in patients treated with ICI compared to those receiving non-ICI active treatment. [46] 26 studies were included. Authors findings suggest that patients who received ICIs report no change in global QOL and have improved QOL compared to patients treated with non-ICI treatments. Global QOL did not change statistically significantly from baseline to follow-up (mean change: 1.13, 95% CI: −0.54 to 2.81). [46] Patients receiving ICIs reported larger improvements in global QOL than patients receiving non-ICI treatments (mean change difference: 3.44, 95% CI: 2.00 to 4.89). [46] Across all ICI regimens, there was no statistically significant change in physical functioning from baseline to follow-up (mean change: 0.46, 95% CI: −0.79 to 1.71). [46]

PROs are considered to be more objective because they rely on patients’ responses instead of subjective assessments by health care providers. [47,48] In the future, baseline PROs could emerge as a better stratification factor than performance status. [37] Also, worsening in PRO scores may correlate with disease progression. [47,49] However, there is a paucity of information in the literature regarding the quality of the data collected from patients during follow-up (i.e., after treatment discontinuation) in clinical trials. [50] In many countries, the follow-up PRO data have been relevant for analyzing comparative effectiveness of new therapies as part of the health technology assessment evaluation process, as well as to determine drug reimbursement and market access. For example, the German Institute for Quality and Efficiency in Health Care requests drug manufacturers to report PRO data collected after disease progression. [51] Although PRO data that are collected after treatment discontinuation can be used to inform various endpoints, the FDA's Oncology Center of Excellence has their primary focus on the use of PROs to inform safety or tolerability while patients are on treatment. [52] Timing and frequency of PRO assessments after treatment discontinuation are critical considerations for meaningful data interpretation. [53] In addition, during cancer clinical trials, the PRO schedule of assessments are generally tied to clinical visits for convenience, despite the possibility that these schedules may not be optimal. The schedule of PRO assessments on treatment versus those in follow-up also varies in time between assessments, making the data difficult to interpret and analyze. Such suboptimal PRO data collection can lead to the potential under- or overestimation of outcomes.

In June 2021, the US FDA issued a draft guidance to outline a set of core PRO measures that can be used in cancer clinical trials. [54] The draft guidance focuses on a set of core PRO measures that can be used to gather data on patients’ symptoms, symptomatic adverse events and physical function, and is specific to registration trials for anti-cancer treatments intended to demonstrate an effect on survival, tumor response or delay in the progression. The US FDA recommends collecting and separately analyzing PROs on disease-related symptoms, adverse events, side effects, physical function, and role function. [54] For example, for disease-related symptoms, the guidance suggests the use of disease symptom scales, such as use of NSCLC Symptom Assessment Questionnaire. [54] The guidance states that, “in contexts where disease symptoms are heterogeneous in type and incidence, symptoms that patients have reported as being important across advanced cancer settings, such as pain, anorexia, and fatigue, can be measured either individually or within a symptom score with other important disease-related symptoms.” For assessing adverse events, the US FDA recommends using the National Cancer Institute's PRO version of the common terminology criteria for adverse events (PRO—CTCAE). [54] However, the PRO—CTCAE may not be sufficiently comprehensive in its current form to incorporate all irAEs.

Since 2015, 21st Century Cures Act helped to bolster the inclusion of PRO measures in clinical trials. [8] But even with an increased emphasis on PRO data, the studies on registrational trials for cancer therapies have shown wide variation in how PRO measures are included and analyzed. A recent study on the registrational trials of multiple myeloma treatments, submitted to the US FDA between January 2007 and January 2020, showed substantial heterogeneity in PRO collection methods, definitions of the patient population being analyzed, completion of the measures, and what constitutes a clinically meaningful change. [55] The authors found that 40 PRO instruments were used across 17 clinical trials. [55] The timing of the PRO assessments were heterogenous, and usually scarce. The analysis showed that the registrational trials also had varying definitions of baseline. For instance, seven trials defined baseline as “cycle 1, day1”, while two trials defined baseline as being “on or prior to cycle 1, day 1″ and eight trials defined baseline as being the “screening phase or before randomization". The trials also used different definitions to show completion of PRO instruments. While one trial defined it as completing all the questions, two trials defined it as completing half of the questions, and 14 trials defined it as “completing enough items to calculate the score in any domain". [55] The authors reported that trials also varied whether they included the intent-to-treat population or a safety population in their analysis.

In another study, the US FDA researchers examined the use of PROs after treatment discontinuation across four solid tumor cancer types: prostate cancer, breast cancer, pancreatic cancer, and hepatocellular carcinoma. [53] They found variation in PRO measure completion rates, and in the duration of follow-up when examining registrational cancer trials for therapies approved by the US FDA between January 2010 and January 2019. [53] The authors reviewed 54 trials, and reported that PRO data were collected for at least one follow-up assessment in 46% of the trials. The schedules for follow-up varied with a first assessment occurring anywhere between 30 days and 6 months after the end of treatment. [53] Mean completion rates for PROs at the first follow-up also varied based on the type of cancer, with completion rates of more than 70% for breast cancer trials and nearly 55% for prostate cancer. PRO completion rates at the first follow up assessment were not available for hepatocellular or pancreatic cancer trials. [53] All in all, the researchers concluded that the follow-up phase of PRO assessments has “not been given the same attention as on-treatment assessments”.

## Conclusion

Nivolumab alleviated symptom burden and improved health status of patients in the registrational clinical trials of advanced NSCLC. However, incapability of the included PRO instruments to measure immune-related AEs, differences in timing of PRO evaluation between treatment groups, incomplete patient participation at all time points, limited patient participation in the later time points, and interpretation of the longitudinal data were posing a compounded challenge to accurately analyze and validate the findings of the clinical trials.

## Funding source

This research did not receive any specific grant from funding agencies in the public, commercial, or not-for-profit sectors.

## CRediT authorship contribution statement

**Remziye Zaim:** Conceptualization, Data curation, Writing – original draft, Writing – review & editing. **Ken Redekop:** Writing – review & editing. **Carin A. Uyl-de Groot:** Writing – review & editing.

## Declaration of Competing Interest

None declared.

## Appendix Supplementary materials

Image, application 1
