# Supplementary material for: Analysis of patient reported outcomes included in the registrational clinical trials of nivolumab for advanced non-small cell lung cancer
Source: Transl Oncol. 2022 Apr 13;20:101418. doi: 10.1016/j.tranon.2022.101418 (PMC9034386; doi:10.1016/j.tranon.2022.101418)
Supplement: Supplementary file 1 [file mmc1.docx]

**Supplementary Appendix**

**Table 1.** Search syntax

| **Category** | **Search terms** |
| --- | --- |
| Population | *“non-small cell cancer” AND (advanced OR metastatic OR recurrent) AND (“PD-1” OR “PD-L1” OR “CTLA-4” OR “programmed cell death” OR “cytotoxic T lymphocytes” )* |
| Intervention | *AND (“phase 3” OR “phase III”) AND (nivolumab OR ipilimumab)* |
| Comparison | N/A (no restriction) |
| Outcome | *AND (qol OR pro OR prom OR "quality of life" OR "patient reported outcome" OR "patient reported outcomes" OR "health-related quality of life")* |
